# Adenocarcinoma in situ and minimally invasive adenocarcinoma in lungs of smokers: image feature differences from those in lungs of non-smokers

**DOI:** 10.1186/s12880-021-00705-1

**Published:** 2021-11-19

**Authors:** Haruto Sugawara, Hirokazu Watanabe, Akira Kunimatsu, Osamu Abe, Shun-ichi Watanabe, Yasushi Yatabe, Masahiko Kusumoto

**Affiliations:** 1 Department of Diagnostic Radiology, National Cancer Center Hospital, 5-1-1 Tsukiji, Chuo-ku, Tokyo, 104-0045 Japan; 2 Department of Radiology, IMSUT Hospital, The Institute of Medical Science, The University of Tokyo, Tokyo, Japan; 3 Department of Radiology, Graduate School of Medicine, The University of Tokyo, Tokyo, Japan; 4 Department of Thoracic Surgery, National Cancer Center Hospital, Tokyo, Japan; 5 Department of Diagnostic Pathology, National Cancer Center Hospital, Tokyo, Japan

## Abstract

**Purpose:**

We aimed to examine the characteristics of imaging findings of adenocarcinoma in situ (AIS) and minimally invasive adenocarcinoma (MIA) in the lungs of smokers compared with those of non-smokers.

**Materials and methods:**

We included seven cases of AIS and 20 cases of MIA in lungs of smokers (pack-years ≥ 20) and the same number of cases of AIS and MIA in lungs of non-smokers (pack-years = 0). We compared the diameter of the entire lesion and solid component measured on computed tomography (CT) images, pathological size and invasive component diameter measured from pathological specimens, and CT values of the entire lesion and ground-glass opacity (GGO) portions between the smoker and non-smoker groups.

**Results:**

The diameters of AIS and MIA on CT images and pathological specimens of the smoker group were significantly larger than those of the non-smoker group (*p* = 0.036 and 0.008, respectively), whereas there was no significant difference in the diameter of the solid component on CT images or invasive component of pathological specimens between the two groups. Additionally, mean CT values of the entire lesion and GGO component of the lesions in the smoker group were significantly lower than those in the non-smoker group (*p* = 0.036 and 0.040, respectively).

**Conclusion:**

AIS and MIA in smoker’s lung tended to have larger lesion diameter and lower internal CT values compared with lesions in non-smoker’s lung. This study calls an attention on smoking status in CT-based diagnosis for early stage adenocarcinoma.

## Introduction

Minimally invasive adenocarcinoma (MIA) and adenocarcinoma in situ (AIS) are subtypes of lung adenocarcinoma as defined by the 2015 WHO classification [1]. These lesions have been shown to have significantly better prognoses than those of invasive adenocarcinoma [2, 3]. Generally, AIS is usually identified as a pure ground-glass nodule (GGN) on computed tomography (CT) images, whereas MIA tends to be identified as a part-solid nodule. Particularly, MIA is defined as a tumor of predominantly lepidic growth with a ≤ 5 mm invasive component on the pathological specimen; therefore, it is typically observed as a part-solid nodule, which is predominantly ground-glass opacity (GGO) with a small solid component in the center [4]. However, these tumors can be challenging to diagnose because of their extremely slow growth rate and consequent lack of significant interval change on CT images [5, 6].

Smoking has been shown to have a considerable impact on background lung status and is considered to be a specific risk factor for emphysema and interstitial lung disease [7, 8]. With the presence of emphysema or interstitial lung disease, histological patterns of lung cancer tend to differ from those of non-smokers; moreover, imaging findings have shown to be different [9, 10]. Specifically, previous reports have shown that CT-based diagnoses of malignant tumors are more difficult in the emphysematous lung compared with the normal lung [11].

As mentioned above, AIS and MIA are generally detected as pure GGNs or part-solid nodules with a predominant GGO portion on CT images. Therefore, it can be hypothesized that background lung changes may significantly affect imaging findings. Because the prognoses of AIS and MIA are good if surgically resected, determining the spectrum of imaging features of AIS and MIA would be valuable to enable early diagnoses based on CT images. To the best of our knowledge, there has not been any report investigating imaging characteristics of AIS and MIA in lungs of smokers. Therefore, this study aimed to examine the characteristics of imaging findings of AIS and MIA in smokers’ lungs compared with those in non-smokers’ lungs.

## Materials and methods

### Study population

All procedures performed in studies involving human participants were in accordance with the ethical standards of the institutional and/or national research committee and with the 1964 Helsinki declaration and its later amendments or comparable ethical standards. This retrospective study was approved by the Institutional Review Board of National Cancer Center Hospital, Tokyo, Japan. The informed consent requirement for this retrospective study was waived by the Institutional Review Board of National Cancer Center Hospital, Tokyo, Japan.

We retrospectively searched for histologically diagnosed cases of AIS and MIA at a single institution between January 2017 and December 2019 and identified 514 cases. Of these, we selected 241 patients with pack-years (number of packs of cigarettes per day × number of years of cigarette smoking) of 0 (non-smoker) and 189 patients with pack-years of 20 or higher [12, 13]. Patients with pack-years of 1–19 were excluded.

AIS and MIA, particularly small AIS, are often identified incidentally, separately from the main lesion on a pathological specimen. Because these incidentally detected and multiple lesions are often small and make CT-pathology comparisons difficult, we only included cases with only one lesion in one surgical specimen. In the smoker group, one case of AIS was excluded from analysis because all parts of the lesion showed soft tissue density on CT images and was therefore considered radiographically atypical. This resulted in 109 cases in the non-smoker group and 27 cases in the smoker group.

The smoker group included seven cases of AIS and 20 cases of MIA. Because we intended to compare average internal CT values between smoker and non-smoker group, which can be significantly affected by the proportion of AIS to MIA lesions, we decided to include the same proportion of AIS to MIA in the non-smoker group. Therefore, we randomly selected seven cases of AIS and 20 cases of MIA from the non-smoker group. None of the 27 cases in the randomly selected non-smoker group showed entire soft tissue density.

**Fig. 1:**
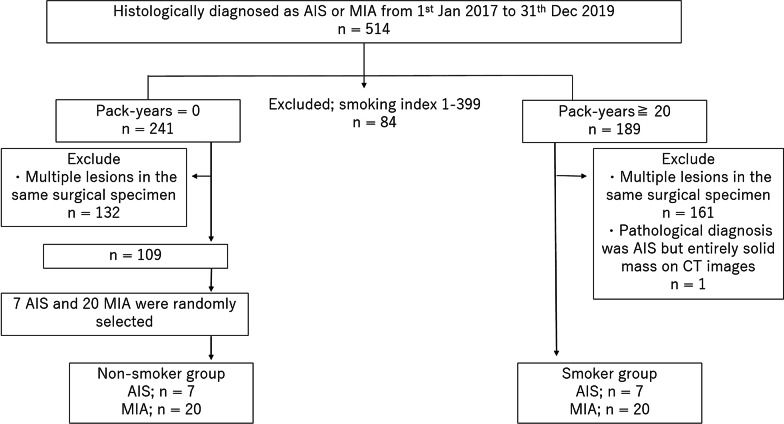
Flowchart of patient inclusion criteria

Figure 1 shows flowchart of patient inclusion criteria.

### CT data acquisition

CT images were acquired on an 80-row or 160-row CT scanner (Aquilion PRIME and Aquilion Precision, Canon Medical Systems, Otawara, Japan). The scan parameters were detector collimation, 0.5 × 80 mm; tube voltage, 120 kVp; pitch, 0.637; gantry rotation, 0.5 s; and scan field of view (FOV), 320–350 mm, depending on patient body size. Auto exposure control was used to determine tube current with a target noise index of 10. A high-frequency reconstruction algorithm was used for lung image reconstruction, with an iterative reconstruction algorithm (AIDR) for denoizing. Images with a reconstructed FOV of 210 mm, focusing on the tumor, and a slice thickness of 1.0 mm were used for tumor analyses.

### Radiological analysis

**Fig. 2:**
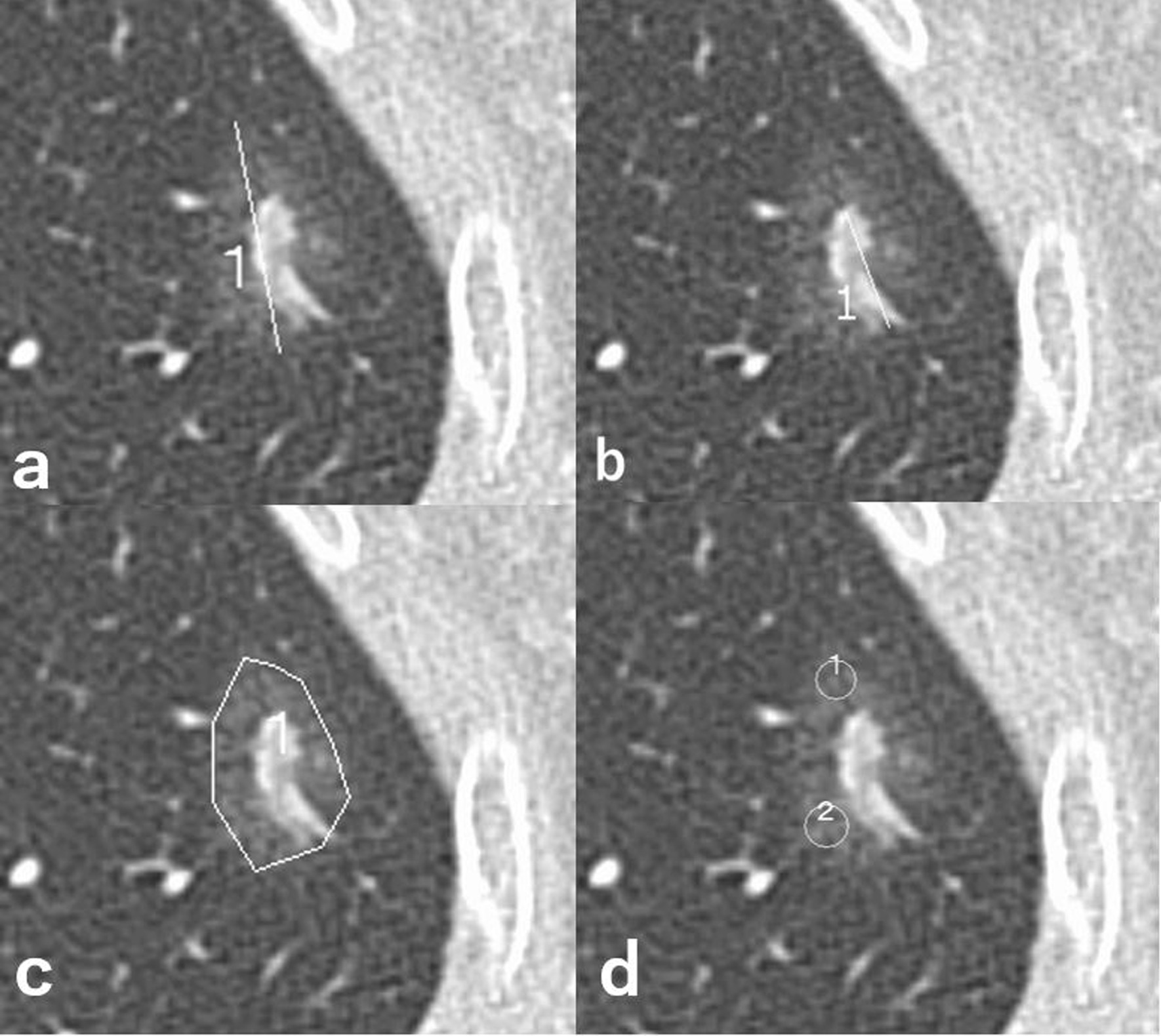
74-year-old non-smoker woman who was pathologically diagnosed with minimally invasive adenocarcinoma. Longest diameter of the entire lesion (**a**), longest diameter of the solid component (**b**), average CT value of the entire lesion (**c**), and average CT value of the ground-glass opacity (GGO) portion on axial slices (**d**)

Two board-certified diagnostic radiologists (reader 1: 8 years of experience; reader 2: 34 years of experience) independently measured the size and density of tumors using commercially available software (ShadeQuest/Report, FUJIFILM Medical Solutions, Tokyo, Japan), being blinded to the clinical information of patients, which included smoking status and pathology. The radiologists first measured the longest diameter of the entire lesion that included the GGO portion (Fig. 2a) and then measured the longest diameter of the solid component (Fig. 2b), which was done in the same way as the routine clinical reporting. If the radiologist concluded that there was no solid component, the diameter of solid component was recorded as 0 mm. They then independently chose the axial slice of the lesion that they thought had the largest proportion of solid components, and surrounded the whole lesion at the determined axial slice, including the GGO portion, with a polygonal region of interest (ROI) and calculated the average CT value inside the ROI (Fig. 2c). Additionally, two circular ROIs of approximately 10 mm^2^ were placed in the GGO area of the lesion, and the CT values inside each were averaged (Fig. 2d). The two ROIs were placed with as little overlap as possible, and were allowed to be placed on different axial slices. All analyses were conducted on axial images with 1.0-mm-slice thickness. The entire lesion diameters, solid component diameters, CT values of the entire lesion, and CT values of the GGO portion measured by the two radiologists were averaged for subsequent analyses.

These values were compared between the non-smoker and smoker groups. Furthermore, we also conducted secondary analyses to compare values between AIS and MIA within each group.

### Pathological diagnosis

AIS and MIA were histologically diagnosed according to the 2015 WHO classification for pulmonary adenocarcinoma [1]. AIS was defined as a purely lepidic-growing small (≤ 3 cm) adenocarcinoma without stromal, vascular, alveolar space, or pleural invasion. MIA was defined as a small (≤ 3 cm) adenocarcinoma with predominantly lepidic growth with a ≤ 5 mm invasive component along the longest dimension without lymphatic, vascular, alveolar space, or pleural invasion.

Pathological tumor size was recorded following the standard procedure. Briefly, the gross sizes of the resected specimens and tumors were noted three-dimensionally. Dimensions were adjusted under a microscope based on sections stained with hematoxylin and eosin. If the tumor had a non-invasive component and/or inflammation, the largest diameter of the invasive component was also measured. All pathological tumor sizes were obtained from pathology reports.

### Statistical analysis

For patient characteristics (age, pack-years, sex, presence of contrast, and histology), significant differences were tested using a chi-square test for categorical variables and a *t*-test for continuous variables. Measurements from CT images and pathological specimens were tested for significance using a non-parametric test (Mann–Whitney *U* Test) because some of the variables were not normally distributed according to the results of the Shapiro–Wilk test.

Statistical analyses were conducted using statistical software R (version 3.6.3). Statistical significance was set at a *p* < 0.05.

## Results

### Patient population

The mean age was 67.7 ± 11.3 years (mean ± standard deviation) for the non-smoker group and 71.8 ± 13.4 for the smoker group, which did not differ significantly between groups. The average pack-years was 39.7 ± 16.2 in the smoker group.

Six out of 27 were men in the non-smoker group, and 20 out of 27 were men in the smoker group; there was a significantly smaller proportion of men in the non-smoker group (*p* < 0.001). Table 1 summarizes the patient information.

**Table 1:** Patient characteristics

	Non-smoker	Smoker	*p*-value
	(n = 27)	(n = 27)	
Age	67.7 ± 11.3	71.8 ± 13.5	*p* = 0.121
pack-years	0	39.7 ± 16.2	*p* < 0.001^*^
Sex			*p* < 0.001^*^
Male	6	20	
Female	21	7	
Contrast material			*p* = 1.000
Enhanced	19	18	
Unenhanced	8	9	
Histological subtype			*p* = 1.000
Adenocarcinoma in situ	7	7	
Minimally invasive adenocarcinoma	20	20	

Values are shown as means ± standard deviations

^*^Significant difference

### Tumor diameter and internal density

Tumor diameter measured from CT images and pathological specimens of the non-smoker group were 14.6 ± 4.7 mm (mean ± standard deviation) and 12.4 ± 4.5 mm, respectively, which were significantly smaller than those of the smoker group (17.1 ± 4.0 mm for CT images, *p* = 0.036 and 16.3 ± 5.5 mm for pathological specimens, *p* = 0.008). However, there was no significant difference in the size of the solid component measured on CT images or the diameter of the invasive component on pathological specimens between the non-smoker and smoker groups.

**Fig. 3:**
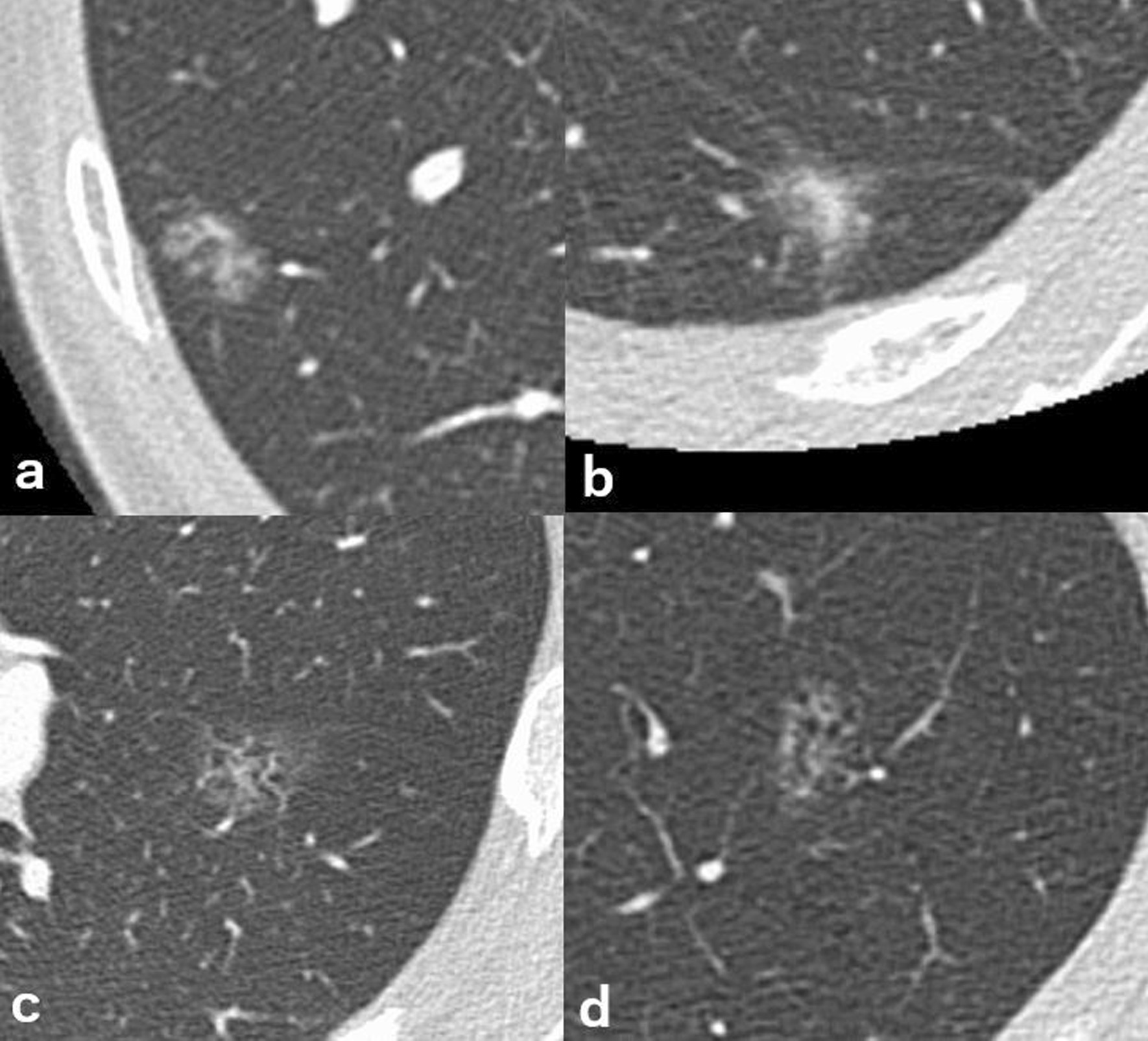
**a** A non-smoker’s MIA with 3 mm invasiveness. **b** A non-smoker’s MIA with 5 mm invasiveness. **c** A smoker’s MIA with 2 mm invasiveness. **d** A smoker’s MIA with 5 mm invasiveness. CT values tended to be lower in lesions arising from smoker’s lungs

The mean CT value of the entire lesion was − 536 ± 105 Hounsfield unit (HU) in the non-smoker group and − 594 ± 78 HU in the smoker group and was significantly lower in the smoker group (*p* = 0.036). The CT value of GGO was also significantly lower in the smoker group (*p* = 0.040): − 549 ± 126 HU in the non-smoking group and − 611 ± 105 HU in the smoking group. Table 2 summarizes the radiological and pathological findings and Fig. 3 shows examples of CT images of lesions.

**Table 2:** Radiological and pathological findings

	Non-smoker	Smoker	*p*-value
	(n = 27)	(n = 27)	
Longest diameter of the entire lesion (mm)			
CT measurement	14.6 ± 4.7	17.1 ± 4.0	*p* = 0.036^*^
Pathological measurement	12.4 ± 4.5	16.3 ± 5.5	*p* = 0.008^*^
Longest diameter of solid/invasive component (mm)			
CT measurement	2.2 ± 2.7	2.4 ± 2.9	*p* = 0.975
Pathological measurement	2.3 ± 1.9	2.1 ± 1.7	*p* = 0.740
Internal CT value (HU)			
CT value of entire lesion	− 536 ± 105	− 594 ± 78	*p* = 0.036^*^
CT value of ground-glass opacity	− 549 ± 126	− 611 ± 105	*p* = 0.040^*^

If the radiologist concluded that there was no solid component, the diameter of solid component was calculated as 0 mm. for pathological measurement, invasive component of AIS was calculated as 0 mm by definition

Values are shown as means ± standard deviations

*HU* Hounsfield unit

^*^Significant difference

Since it has been reported that contrast material administration can affect the measurement of tumor diameters and CT values [14, 15], we conducted a subgroup analysis by dividing the population into either unenhanced or contrast-enhanced. Overall, a similar trend was observed in this subgroup analysis. Although not statistically significant, the CT value of the entire lesion and the CT value of the ground-glass opacity tended to be lower in the smoker group for both the unenhanced and contrast-enhanced subgroups. In the contrast-enhanced subgroup, the longest diameter of the entire lesion was significantly larger in the smoker group for both the CT and pathological measurements (*p* = 0.029 and *p* < 0.001, respectively). However, no significant difference was observed in the unenhanced subgroup. These results are summarized in Table 3.

**Table 3:** Subgroup analysis based on unenhanced and contrast-enhanced CT

Unenhanced examination	Non-smoker	Smoker	*p*-value
	(n = 8)	(n = 9)	
Longest diameter of the entire lesion (mm)			
CT measurement	16.3 ± 4.1	17.1 ± 5.4	*p* = 0.888
Pathological measurement	15.0 ± 5.2	17.3 ± 8.1	*p* = 1.000
Longest diameter of solid/invasive component (mm)			
CT measurement	2.3 ± 3.0	2.8 ± 2.6	*p* = 0.807
Pathological measurement	2.2 ± 1.7	2.0 ± 1.6	*p* = 0.799
Internal CT value (HU)			
CT value of entire lesion	− 569 ± 43	− 603 ± 72	*p* = 0.265
CT value of ground-glass opacity	− 550 ± 82	− 623 ± 83	*p* = 0.093

Contrast-enhanced examination	Non-smoker	Smoker	*p*-value
	(n = 19)	(n = 18)	
Longest diameter of the entire lesion (mm)			
CT measurement	13.9 ± 4.9	17.2 ± 3.3	*p* = 0.029^*^
Pathological measurement	11.4 ± 3.7	15.7 ± 3.7	*p* < 0.001^*^
Longest diameter of solid/invasive component (mm)			
CT measurement	2.2 ± 2.6	2.2 ± 3.1	*p* = 0.834
Pathological measurement	2.3 ± 2.1	2.2 ± 1.8	*p* = 0.849
Internal CT value (HU)			
CT value of entire lesion	− 523 ± 121	− 590 ± 82	*p* = 0.096
CT value of ground-glass opacity	− 550 ± 142	− 606 ± 116	*p* = 0.196

If the radiologist concluded that there was no solid component, the diameter of solid component was calculated as 0 mm. for pathological measurement, invasive component of AIS was calculated as 0 mm by definition

Values are shown as means ± standard deviations

*HU* Hounsfield unit

^*^Significant difference

### Secondary analyses by histological subtypes

In the non-smoker group, the CT value of the entire lesion for AIS was − 612 ± 87 HU, which was significantly lower than that for MIA, which was − 510 ± 100 HU. By contrast, in the smoker group, the CT value for AIS was − 596 ± 62 HU and for MIA was − 594 ± 84 HU, which was not significantly different. The longest diameter of the solid/invasive component (mm) on the CT image of MIA was significantly larger (2.9 ± 2.8 mm) than that of AIS (0.2 ± 0.4 mm) in the non-smoker group (*p* = 0.012). However, no significant difference was observed in the smoker group (1.7 ± 2.7 mm for AIS; and 2.6 ± 3.0 mm for MIA, *p* = 0.547). The longest diameter of the entire lesion (mm) on the CT image was not significantly different between AIS and MIA in both the smoker and non-smoker groups.

**Fig. 4:**
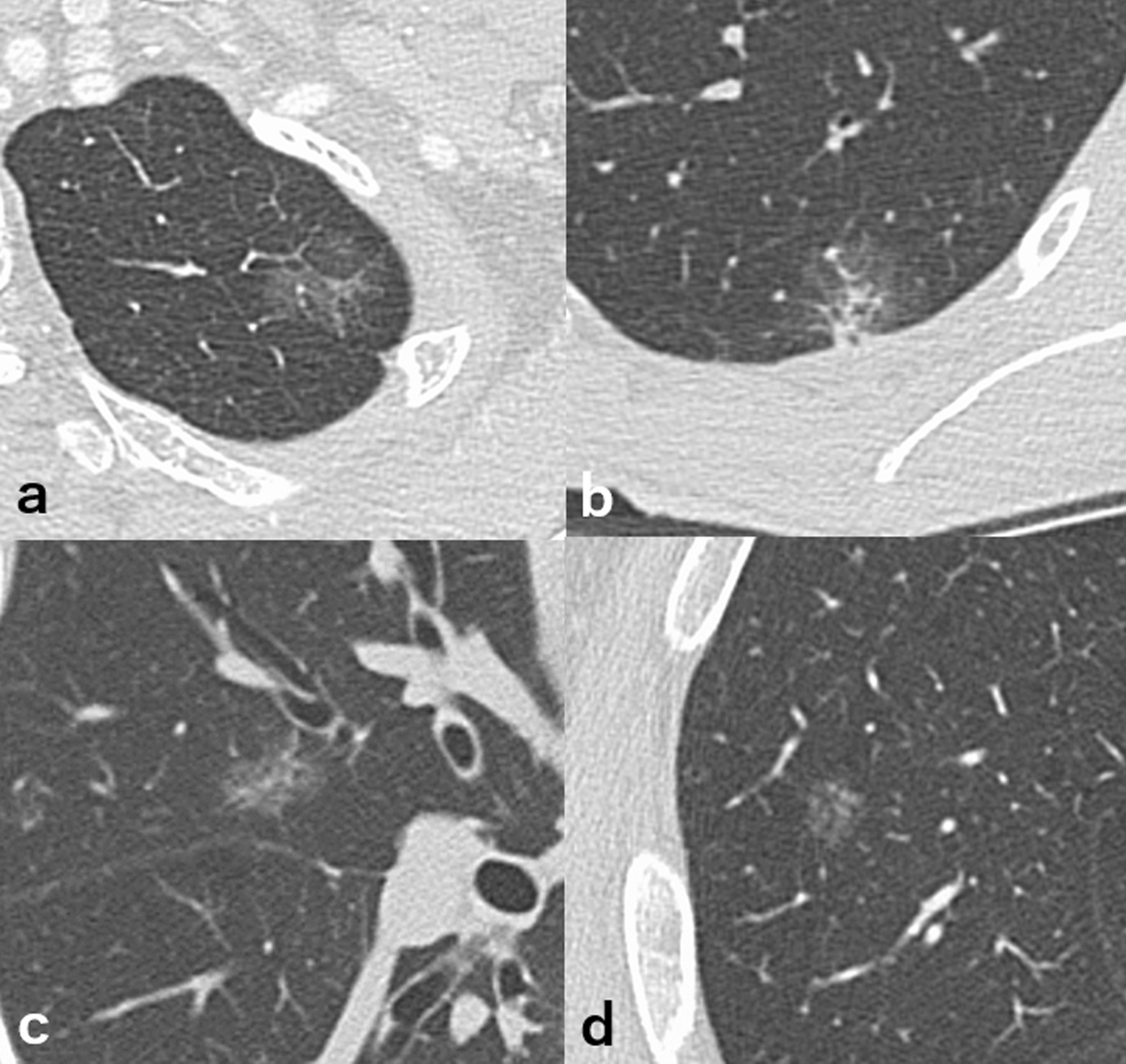
Adenocarcinoma in situ (AIS) (**a**) and minimally invasive adenocarcinoma (MIA) (**b**) arising from a non-smoker’s lung. The MIA arising from a non-smoker’s lung shows relatively typical radiological findings with GGO at the periphery and a solid component in the center. Conversely, AIS (**c**) and MIA (**d**) arising from a smoker’s lung show relatively low density and are difficult to differentiate

Table 4 summarizes these results. Figure 4 shows examples of CT images of lesions.

**Table 4:** CT values of the entire lesion (HU) for adenocarcinoma in situ (AIS) and minimally invasive adenocarcinoma (MIA)

	Non-smoker	Smoker
	(n = 27)	(n = 27)
CT value of entire lesion		
Adenocarcinoma in situ (AIS) (n = 7)	− 612 ± 87	− 596 ± 62
Minimally invasive adenocarcinoma (MIA) (n = 20)	− 510 ± 100	− 594 ± 84
*p*-values	*p* = 0.018*	*p* = 0.901
Longest diameter of the entire lesion (mm) on CT image		
Adenocarcinoma in situ (AIS) (n = 7)	12.6 ± 5.8	17.0 ± 3.4
Minimally invasive adenocarcinoma (MIA) (n = 20)	15.3 ± 4.2	17.2 ± 4.3
*p*-values	*p* = 0.16	*p* = 1.000
Longest diameter of solid/invasive component (mm) on CT image		
Adenocarcinoma in situ (AIS) (n = 7)	0.2 ± 0.4	1.7 ± 2.7
Minimally invasive adenocarcinoma (MIA) (n = 20)	2.9 ± 2.8	2.6 ± 3.0
*p*-values	*p* = 0.012*	*p* = 0.547

^*^Significant difference

## Discussion

In this study, AIS and MIA arising from smoker’s lung had larger diameter, higher proportion of GGO portions, and lower internal CT value compared with those arising from non-smoker’s lung.

Typically, AIS shows pure GGN on CT images and MIA appears as a part-solid nodule [4]. Although MIA has an invasive component, prognosis after resection is good [2, 3]; thus, early diagnosis of MIA is considered extremely important. Several previous studies have reported that the invasiveness of lung adenocarcinomas can be differentiated by internal CT values [16, 17]. In other words, the extent of invasion is represented by the solid components on CT and higher internal CT values in MIA and adenocarcinoma. However, we found that AIS and MIA arising from smoker’s lung tended to have lower internal CT values than those arising from non-smoker’s lung. Therefore, when GGO or part-solid GGO is present in a smoker’s lung, differentiation of invasiveness based on internal CT values alone may be misleading. Additionally, AIS and MIA in the lungs of smokers had significantly larger diameters than those in the lungs of non-smokers; however, there were no significant differences in solid component size on CT or invasiveness diameter on pathological specimens. Although it has been reported that contrast material administration can affect the measurement of tumor diameters and CT values [14, 15], we observed a similar trend in the subgroup analysis based on the contrast material administration. In the subgroup analysis, the CT values in the smoker group tended to be lower than those in the non-smoker group, but the p-value was not significant. This may be due to the lower detection power caused by the smaller sample size in the subgroup analysis.

Although previous studies have shown that invasiveness of early-stage adenocarcinomas can be distinguished by the ratio of solid portion to GGO portion [18, 19], our result suggested that smoking status also affects this ratio and should be taken into account when making CT-based diagnoses.

Furthermore, as reported previously, average CT values were significantly smaller in AIS than in MIA in non-smoker’s lungs [16, 17]. Surprisingly, in the present study, there was no significant difference in average CT values between the two histological subtypes in the smoker group, which demonstrated that differentiating AIS from MIA based on CT values is challenging in smoker’s lungs. Additionally, in line with previous studies, the longest diameter of the solid/invasive component on the CT image of MIA was significantly larger than that of AIS in the non-smoker group, but no significant difference was observed in the smoker group, which could make CT-based differentiation of AIS and MIA even more challenging. A possible explanation may be that AIS and MIA in the smoker group tended to have a larger proportion of GGO, which may result in lower overall CT values. Additionally, previous reports have shown that the solid part on CT images does not always reflect cancer cells but may reflect a fibroblastic change or alveolar collapse [20, 21]. In smoker’s lungs, it may be possible that these non-neoplastic solid components are more frequently observed in AIS because of chronic inflammation.

Several factors may contribute to these atypical imaging features of AIS and MIA that occur in smoker’s lungs. First, emphysematous changes often occur in the lungs of smokers. Even with mild changes that are less detectable on CT images, the density of tumor cells showing lepidic growth may be decreased because of the enlargement of air space and destruction of alveolar structures [22]. In fact, in the present study, it was not only the CT value of the entire lesion that was lower in smokers compared with non-smokers but also the CT value of the GGO portion. This suggests that the background lung status itself may affect CT values of lesions showing lepidic growth.

Genetic factors may also explain the imaging features of AIS and MIA that develop in smoker’s lungs. Previous studies have suggested that AAH, AIS, and MIA that occur in smoker’s lungs may be associated with mutations of the *KRAS* gene, whereas those occurring in non-smoker’s lungs may be associated with *EGFR* mutations [23, 24]. These genetic features have also been associated with the tendency for lesions to increase in size [25]. In fact, a previous study has shown that GGOs identified on CT images are more likely to increase in the lungs of smokers than in those of non-smokers [6]. In the present study, the overall diameter, which included the GGO portion of the lesion, tended to be larger in lesions arising from smoker’s lung, and it may be possible that the differences in imaging features reflect different genetic characteristics.

There were several limitations to this study. Firstly, this was a single center retrospective study, which could incur selection bias, and therefore the results cannot be overgeneralized. Secondly, as for multiple lesions, many lesions were found incidentally on pathological specimens and could not be identified by CT due to their extremely small size. For this reason, we excluded multiple lesions from our analysis, which resulted in a relatively small number of cases. Thirdly, in this study, only lesions that were surgically resected were included. The results of the present study suggest that the internal density on CT images of AIS and MIA occurring in smoker’s lung tends to be low. If this is influenced by potential emphysematous changes, AIS and MIA arising from severe and advanced emphysematous lung, may not have been included in the current study because of the difficulty in diagnosing the presence of the lesions themselves.

In conclusion, AIS and MIA arising from smoker’s lung tended to have a larger lesion diameter and lower internal CT values compared with lesions arising from non-smoker’s lungs.

## Data Availability

The datasets generated and/or analyzed during the current study are not publicly available due to their containing information that could compromise patient privacy and confidentiality, but are available from the corresponding author on reasonable request.

## Abbreviations

MIA: Minimally invasive adenocarcinoma
AIS: Adenocarcinoma in situ
GGN: Ground-glass nodule
CT: Computed tomography
GGO: Ground-glass opacity
FOV: Field of view
ROI: Region of interest
HU: Hounsfield unit

## References

[1] Travis WD, Brambilla E, Burke A (2015). WHO classification of Tumours of the lung, pleura, thymus and heart. Lyon: international agency for research on cancer. J Thorac Oncol.

[2] Yoshizawa A, Motoi N, Riely GJ, Sima CS, Gerald WL, Kris MG, Park BJ, Rusch VW, Travis WD (2011). Impact of proposed IASLC/ATS/ERS classification of lung adenocarcinoma: prognostic subgroups and implications for further revision of staging based on analysis of 514 stage I cases. Mod Pathol.

[3] Ishida H, Shimizu Y, Sakaguchi H, Nitanda H, Kaneko K, Yamazaki N, Yanagihara A, Taguchi R, Sakai F, Yasuda M, Kobayashi K (2019). Distinctive clinicopathological features of adenocarcinoma in situ and minimally invasive adenocarcinoma of the lung: a retrospective study. Lung Cancer.

[4] Lee KH, Goo JM, Park SJ, Wi JY, Chung DH, Go H, Park HS, Park CM, Lee SM (2014). Correlation between the size of the solid component on thin-section CT and the invasive component on pathology in small lung adenocarcinomas manifesting as ground-glass nodules. J Thorac Oncol.

[5] Chang B, Hwang JH, Choi YH, Chung MP, Kim H, Kwon OJ, Lee HY, Lee KS, Shim YM, Han J, Um SW (2013). Natural history of pure ground-glass opacity lung nodules detected by low-dose CT scan. Chest.

[6] Kobayashi Y, Sakao Y, Deshpande GA, Fukui T, Mizuno T, Kuroda H, Sakakura N, Usami N, Yatabe Y, Mitsudomi T (2014). The association between baseline clinical-radiological characteristics and growth of pulmonary nodules with ground-glass opacity. Lung Cancer.

[7] Remy-Jardin M, Remy J, Boulenguez C, Sobaszek A, Edme JL, Furon D (1993). Morphologic effects of cigarette smoking on airways and pulmonary parenchyma in healthy adult volunteers: CT evaluation and correlation with pulmonary function tests. Radiology.

[8] Washko GR, Hunninghake GM, Fernandez IE, Nishino M, Okajima Y, Yamashiro T, Ross JC, Estépar RS, Lynch DA, Brehm JM, Andriole KP, Diaz AA, Khorasani R, D’Aco K, Sciurba FC, Silverman EK, Hatabu H, Rosas IO (2011). COPD gene investigators. Lung volumes and emphysema in smokers with interstitial lung abnormalities. N Engl J Med..

[9] Smith BM, Schwartzman K, Kovacina B, Taylor J, Kasymjanova G, Brandao G, Agulnik JS (2012). Lung cancer histologies associated with emphysema on computed tomography. Lung Cancer.

[10] Masai K, Tsuta K, Motoi N, Shiraishi K, Furuta K, Suzuki S, Asakura K, Nakagawa K, Sakurai H, Watanabe SI, Hiraoka N, Asamura H (2016). Clinicopathological, immunohistochemical, and genetic features of primary lung adenocarcinoma occurring in the setting of usual interstitial pneumonia pattern. J Thorac Oncol.

[11] Matsuoka S, Kurihara Y, Yagihashi K, Niimi H, Nakajima Y (2005). Peripheral solitary pulmonary nodule: CT findings in patients with pulmonary emphysema. Radiology.

[12] Nagata N, Niikura R, Shimbo T, Kishida Y, Sekine K, Tanaka S, Aoki T, Watanabe K, Akiyama J, Yanase M, Itoh T, Mizokami M, Uemura N (2013). Alcohol and smoking affect risk of uncomplicated colonic diverticulosis in Japan. PLOS ONE.

[13] Pinsky PF, Kramer BS (2015). Lung cancer risk and demographic characteristics of current 20–29 pack-year smokers: implications for screening. J Natl Cancer Inst.

[14] Gao F, Li M, Sun Y, Xiao L, Hua Y (2017). Diagnostic value of contrast-enhanced CT scans in identifying lung adenocarcinomas manifesting as GGNs (ground glass nodules). Medicine (Baltimore).

[15] Rampinelli C, Raimondi S, Padrenostro M, De Fiori E, Meroni S, Veronesi G, Bellomi M (2010). Pulmonary nodules: contrast-enhanced volumetric variation at different CT scan delays. AJR Am J Roentgenol.

[16] Ichinose J, Kawaguchi Y, Nakao M, Matsuura Y, Okumura S, Ninomiya H, Oikado K, Nishio M, Mun M (2020). Utility of maximum CT value in predicting the invasiveness of pure ground-glass nodules. Clin Lung Cancer.

[17] Kitami A, Sano F, Hayashi S, Suzuki K, Uematsu S, Kamio Y, Suzuki T, Kadokura M, Omatsu M, Kunimura T (2016). Correlation between histological invasiveness and the computed tomography value in pure ground-glass nodules. Surg Today.

[18] Lee SM, Park CM, Goo JM, Lee HJ, Wi JY, Kang CH (2013). Invasive pulmonary adenocarcinomas versus preinvasive lesions appearing as ground-glass nodules: differentiation by using CT features. Radiology.

[19] Yanagawa M, Kusumoto M, Johkoh T, Noguchi M, Minami Y, Sakai F, Asamura H, Tomiyama N (2018). Investigators of JSTR lung cancer working group. Radiologic-pathologic correlation of solid portions on thin-section CT images in lung adenocarcinoma: a multicenter study. Clin Lung Cancer.

[20] Noguchi M (2010). Stepwise progression of pulmonary adenocarcinoma–clinical and molecular implications. Cancer Metastasis Rev.

[21] Noguchi M, Morikawa A, Kawasaki M, Matsuno Y, Yamada T, Hirohashi S, Kondo H, Shimosato Y (1995). Small adenocarcinoma of the lung. Histol Charact Progn Cancer.

[22] Tanabe N, Vasilescu DM, Hague CJ, Ikezoe K, Murphy DT, Kirby M, Stevenson CS, Verleden SE, Vanaudenaerde BM, Gayan-Ramirez G, Janssens W, Coxson HO, Paré PD, Hogg JC (2020). Pathological Comparisons of paraseptal and centrilobular emphysema in chronic obstructive pulmonary disease. Am J Respir Crit Care Med.

[23] Zhang YL, Yuan JQ, Wang KF, Fu XH, Han XR, Threapleton D, Yang ZY, Mao C, Tang JL (2016). The prevalence of EGFR mutation in patients with non-small cell lung cancer: a systematic review and meta-analysis. Oncotarget.

[24] Mao C, Qiu LX, Liao RY, Du FB, Ding H, Yang WC, Li J, Chen Q (2010). KRAS mutations and resistance to EGFR-TKIs treatment in patients with non-small cell lung cancer: a meta-analysis of 22 studies. Lung Cancer.

[25] Kobayashi Y, Mitsudomi T, Sakao Y, Yatabe Y (2015). Genetic features of pulmonary adenocarcinoma presenting with ground-glass nodules: the differences between nodules with and without growth. Ann Oncol.

